# *N*,*N*′-Bis(2-thienylmethyl­ene)benzene-1,4-diamine

**DOI:** 10.1107/S1600536809031869

**Published:** 2009-08-19

**Authors:** Nai-Wei Dong, Dong-Xue Jia, Chun-Li Gan, Dong-Mei Zhou, Feng-Zhi Liu

**Affiliations:** a School of Pharmaceutical Science, Harbin Medical University, Harbin 150086, People’s Republic of China

## Abstract

The Schiff base, C_16_H_12_N_2_S_2_, has been synthesized by refluxing an ethano­lic solution of thio­phene-2-carbaldehyde and benzene-1,4-diamine. The center of the benzene ring is located on a crystallographic center of inversion. The dihedral angle between the benzene and thio­phene rings is 63.6 (1)°.

## Related literature

For general background to Schiff base complexes, see: Andersen *et al.* (2005 ▶); Koizumi *et al.* (2005 ▶); Boskovic *et al.* (2003 ▶); Oshio *et al.* (2005 ▶). For the synthesis, see: Kannappan *et al.* (2005 ▶).
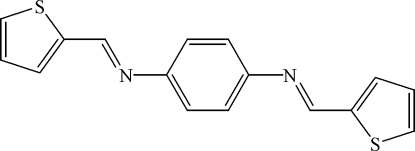


## Experimental

### 

#### Crystal data

C_16_H_12_N_2_S_2_
*M* *_r_* = 296.40
Monoclinic, 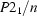

*a* = 6.1882 (7) Å
*b* = 7.2371 (9) Å
*c* = 16.375 (2) Å
β = 95.860 (2)°
*V* = 729.5 (2) Å^3^
*Z* = 2
Mo *K*α radiation
μ = 0.36 mm^−1^
*T* = 294 K
0.15 × 0.11 × 0.09 mm

#### Data collection

Bruker APEXII CCD area-detector diffractometer
Absorption correction: multi-scan (*SADABS*; Sheldrick, 2004 ▶) *T*_min_ = 0.949, *T*_max_ = 0.969
3380 measured reflections
1252 independent reflections
1021 reflections with *I* > 2σ(*I*)
*R*_int_ = 0.019

#### Refinement

*R*[*F* ^2^ > 2σ(*F* ^2^)] = 0.065
*wR*(*F* ^2^) = 0.196
*S* = 1.00
1252 reflections
91 parameters
H-atom parameters not refined
Δρ_max_ = 1.07 e Å^−3^
Δρ_min_ = −0.21 e Å^−3^

### 

Data collection: *APEX2* (Bruker, 2002 ▶); cell refinement: *SAINT-Plus* (Bruker, 2003 ▶); data reduction: *SAINT-Plus*; program(s) used to solve structure: *SHELXTL* (Sheldrick, 2008 ▶); program(s) used to refine structure: *SHELXTL*; molecular graphics: *SHELXTL*; software used to prepare material for publication: *SHELXTL*.

## Supplementary Material

Crystal structure: contains datablocks I, global. DOI: 10.1107/S1600536809031869/im2135sup1.cif

Structure factors: contains datablocks I. DOI: 10.1107/S1600536809031869/im2135Isup2.hkl

Additional supplementary materials:  crystallographic information; 3D view; checkCIF report

## Figures and Tables

**Fig. 1.**
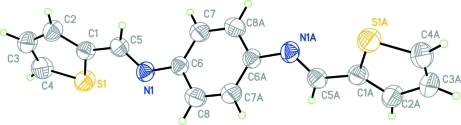
View of the molecular structure of the title compound, showing the atomic numbering scheme and 50% probability displacement ellipsoids. Atoms labeled with A at the symmetry positions (-x + 1,-y + 1,-z + 1).

## supplementary crystallographic information

### Comment

During the past decades, Schiff bases have been intensively investigated not only because of their strong coordination capability but also due to diverse biological activities, such as e.g. antibacterial and antitumor activities (Andersen *et al.*, 2005; Koizumi *et al.*, 2005; Boskovic *et al.*, 2003; Oshio *et al.*, 2005). Here we report the structure of N,N'-bis-thiophene-2-yl-methylene-1,4-benzenediamine, which could be used as another Schiff base ligand in combination with metal ions.

The molecular structure of the title compound is depicted in Figure 1. The center of the phenyl ring represents a crystallographic center of inversion. The dihedral angle between the phenyl and thiophene rings is 63.6 (1)°. As it is expected the thiophene rings also are perfectly planar with a deviation of the S atom of 0.001 Å.

### Experimental

N,N'-Bis-thiophene-2-yl-methylene-1,4-benzenediamine was prepared according to the method reported in the literature (Kannappan *et al.*, 2005). Thiophene-2-carboxaldehyde (0.02 mol) was added to a stirred ethanolic solution of *p*-phenylenediamine (0.01 mol). The reaction mixture was stirred about 3 h and then the mixture was allowed to stand at room temperature for about two days. Yellow crystals suitable for X-ray diffraction analysis were collected with a yield of 60%. Anal. Calc. for C_16_H_12_N_2_S_2_: C 64.78, H 4.05, N 9.45%; Found: C 64.59, H 4.01, N 9.25%.

### Refinement

All H atoms were placed in calculated positions with C—H = 0.93Å and refined as riding with *U*_iso_(H) = 1.2*U*_eq_(carrier).

### Crystal data

**Table:**

C_16_H_12_N_2_S_2_	*F*(000) = 308
*M**_r_* = 296.40	*D*_x_ = 1.349 Mg m^−^^3^
Monoclinic, *P*2_1_/*n*	Mo *K*α radiation, λ = 0.71073 Å
Hall symbol: -P 2yn	Cell parameters from 1252 reflections
*a* = 6.1882 (7) Å	θ = 3.1–25.0°
*b* = 7.2371 (9) Å	µ = 0.36 mm^−^^1^
*c* = 16.375 (2) Å	*T* = 294 K
β = 95.860 (2)°	Block, yellow
*V* = 729.5 (2) Å^3^	0.15 × 0.11 × 0.09 mm
*Z* = 2	

### Data collection

**Table:**

Bruker APEXII CCD area-detector diffractometer	1252 independent reflections
Radiation source: fine-focus sealed tube	1021 reflections with *I* > 2σ(*I*)
graphite	*R*_int_ = 0.019
φ and ω scans	θ_max_ = 25.0°, θ_min_ = 3.1°
Absorption correction: multi-scan (*SADABS*; Sheldrick, 2004)	*h* = −7→7
*T*_min_ = 0.949, *T*_max_ = 0.969	*k* = −8→8
3380 measured reflections	*l* = −19→10

### Refinement

**Table:**

Refinement on *F*^2^	Primary atom site location: structure-invariant direct methods
Least-squares matrix: full	Secondary atom site location: difference Fourier map
*R*[*F*^2^ > 2σ(*F*^2^)] = 0.065	Hydrogen site location: inferred from neighbouring sites
*wR*(*F*^2^) = 0.196	H-atom parameters not refined
*S* = 1.00	*w* = 1/[σ^2^(*F*_o_^2^) + (0.141*P*)^2^ + 0.3146*P*] where *P* = (*F*_o_^2^ + 2*F*_c_^2^)/3
1252 reflections	(Δ/σ)_max_ < 0.001
91 parameters	Δρ_max_ = 1.07 e Å^−^^3^
0 restraints	Δρ_min_ = −0.21 e Å^−^^3^

### Special details

**Table:**

Geometry. All e.s.d.'s (except the e.s.d. in the dihedral angle between two l.s. planes) are estimated using the full covariance matrix. The cell e.s.d.'s are taken into account individually in the estimation of e.s.d.'s in distances, angles and torsion angles; correlations between e.s.d.'s in cell parameters are only used when they are defined by crystal symmetry. An approximate (isotropic) treatment of cell e.s.d.'s is used for estimating e.s.d.'s involving l.s. planes.
Refinement. Refinement of *F*^2^ against ALL reflections. The weighted *R*-factor *wR* and goodness of fit *S* are based on *F*^2^, conventional *R*-factors *R* are based on *F*, with *F* set to zero for negative *F*^2^. The threshold expression of *F*^2^ > σ(*F*^2^) is used only for calculating *R*-factors(gt) *etc*. and is not relevant to the choice of reflections for refinement. *R*-factors based on *F*^2^ are statistically about twice as large as those based on *F*, and *R*- factors based on ALL data will be even larger.

### Fractional atomic coordinates and isotropic or equivalent isotropic displacement parameters (Å^2^)

**Table:**

	*x*	*y*	*z*	*U*_iso_*/*U*_eq_	
S1	0.15131 (14)	−0.10612 (13)	0.29041 (6)	0.0619 (5)	
N1	0.3196 (4)	0.1917 (4)	0.41314 (16)	0.0486 (7)	
C1	0.0201 (5)	0.0013 (4)	0.36497 (18)	0.0462 (7)	
C2	−0.1804 (5)	−0.0755 (5)	0.3705 (2)	0.0565 (9)	
H2	−0.2774	−0.0333	0.4061	0.068*	
C3	−0.2234 (6)	−0.2258 (5)	0.3163 (3)	0.0664 (10)	
H3	−0.3489	−0.2972	0.3136	0.080*	
C4	−0.0594 (6)	−0.2534 (6)	0.2685 (3)	0.0707 (11)	
H4	−0.0624	−0.3437	0.2280	0.085*	
C5	0.1188 (5)	0.1553 (4)	0.41151 (18)	0.0456 (7)	
H5	0.0321	0.2297	0.4411	0.055*	
C6	0.4043 (5)	0.3492 (4)	0.45720 (17)	0.0422 (7)	
C7	0.3093 (5)	0.5209 (5)	0.4490 (2)	0.0564 (9)	
H7	0.1820	0.5361	0.4142	0.068*	
C8	0.6000 (6)	0.3299 (5)	0.5086 (2)	0.0576 (9)	
H8	0.6681	0.2153	0.5138	0.069*	

### Atomic displacement parameters (Å^2^)

**Table:**

	*U*^11^	*U*^22^	*U*^33^	*U*^12^	*U*^13^	*U*^23^
S1	0.0552 (6)	0.0657 (7)	0.0656 (7)	−0.0030 (4)	0.0105 (4)	−0.0170 (4)
N1	0.0464 (15)	0.0470 (15)	0.0525 (15)	−0.0058 (11)	0.0059 (11)	−0.0114 (12)
C1	0.0453 (16)	0.0484 (17)	0.0436 (15)	0.0007 (12)	−0.0013 (12)	−0.0027 (13)
C2	0.0483 (18)	0.062 (2)	0.059 (2)	−0.0059 (15)	0.0054 (15)	−0.0085 (16)
C3	0.0528 (19)	0.061 (2)	0.083 (3)	−0.0100 (16)	−0.0059 (18)	−0.0154 (19)
C4	0.068 (2)	0.063 (2)	0.079 (2)	−0.0017 (18)	−0.0038 (19)	−0.0248 (19)
C5	0.0500 (17)	0.0447 (16)	0.0424 (16)	0.0006 (13)	0.0052 (13)	−0.0041 (12)
C6	0.0422 (16)	0.0427 (15)	0.0417 (15)	−0.0024 (11)	0.0046 (12)	−0.0051 (12)
C7	0.0480 (17)	0.0531 (19)	0.065 (2)	0.0017 (14)	−0.0066 (15)	−0.0032 (16)
C8	0.055 (2)	0.0484 (18)	0.068 (2)	0.0073 (14)	−0.0004 (16)	−0.0039 (16)

### Geometric parameters (Å, °)

**Table:**

S1—C4	1.694 (4)	C3—H3	0.9300
S1—C1	1.719 (3)	C4—H4	0.9300
N1—C5	1.268 (4)	C5—H5	0.9300
N1—C6	1.421 (4)	C6—C7	1.375 (4)
C1—C2	1.371 (4)	C6—C8	1.410 (4)
C1—C5	1.449 (4)	C7—C8^i^	1.373 (5)
C2—C3	1.412 (5)	C7—H7	0.9300
C2—H2	0.9300	C8—C7^i^	1.373 (5)
C3—C4	1.358 (6)	C8—H8	0.9300
			
C4—S1—C1	91.48 (17)	S1—C4—H4	123.5
C5—N1—C6	119.1 (3)	N1—C5—C1	122.0 (3)
C2—C1—C5	127.8 (3)	N1—C5—H5	119.0
C2—C1—S1	111.1 (2)	C1—C5—H5	119.0
C5—C1—S1	121.1 (2)	C7—C6—C8	118.8 (3)
C1—C2—C3	112.6 (3)	C7—C6—N1	122.9 (3)
C1—C2—H2	123.7	C8—C6—N1	118.2 (3)
C3—C2—H2	123.7	C8^i^—C7—C6	120.8 (3)
C4—C3—C2	111.8 (3)	C8^i^—C7—H7	119.6
C4—C3—H3	124.1	C6—C7—H7	119.6
C2—C3—H3	124.1	C7^i^—C8—C6	120.4 (3)
C3—C4—S1	113.0 (3)	C7^i^—C8—H8	119.8
C3—C4—H4	123.5	C6—C8—H8	119.8

Symmetry codes: (i) −*x*+1, −*y*+1, −*z*+1.
